# Gastric Perforation Necessitating Splenectomy

**DOI:** 10.7759/cureus.71091

**Published:** 2024-10-08

**Authors:** Kalvin Zee, Maianh Tran, Noah Pirozzi, Joseph Losh

**Affiliations:** 1 Emergency General Surgery, MercyOne Des Moines Medical Center, Des Moines, USA

**Keywords:** enteric pills, foreign body, gastric ulcer perforation, modified graham's patch, total splenectomy

## Abstract

Gastric perforations can rarely cause splenic erosion from irritation of gastric contents spilling into the peritoneum. This case describes a 51-year-old female on chronic steroid use due to Addison’s disease who presents with a gastric perforation involving the splenic hilum. Computed tomography showed a posterior gastric perforation with splenic erosion of a foreign body. Due to her abdominal exam, surgery for abdominal peritonitis was performed. The patient was found to have a pill that had eroded into the splenic hilum after passing through a gastric perforation. This required concomitant Graham patch repair and splenectomy.

## Introduction

Gastric perforations are full-thickness injuries of the stomach wall, which can create communication between the enteric contents of the stomach and the peritoneal cavity, leading to irritation of surrounding structures [1]. These perforations are commonly due to peptic ulcer disease secondary to non-steroidal anti-inflammatory drugs (NSAIDs), corticosteroid use, or excessive alcohol use, causing chemical peritonitis as the contents leak into the peritoneum [1]. Historically, NSAID use was the most common cause of gastric perforation but has subsequently decreased due to the introduction of proton pump inhibitors [1]. Rarely, undigested enteric contents, such as pills, can escape into the peritoneum, damaging surrounding structures [2]. We describe a case of gastric perforation allowing enteric pills to irritate the splenic hilum, necessitating a concomitant splenectomy with a Graham patch repair.

## Case presentation

**Figure 1:**
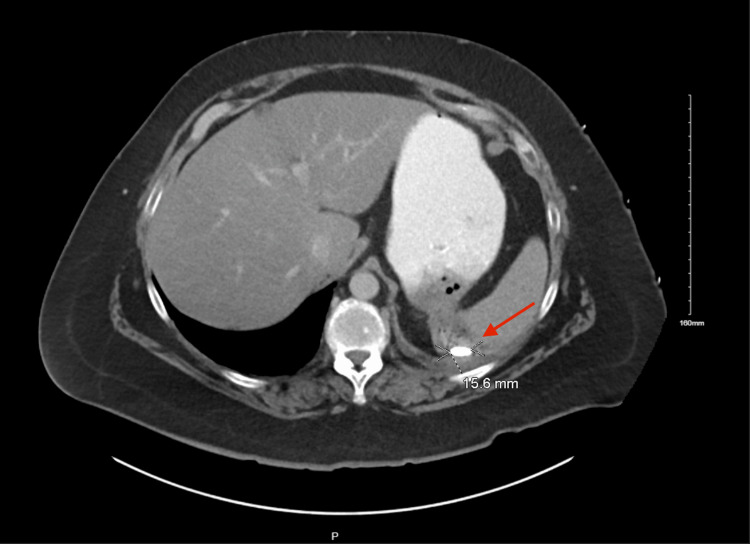
Axial view of perforated gastric ulcer and contiguous splenic erosion with a foreign body posteriorly

**Figure 2:**
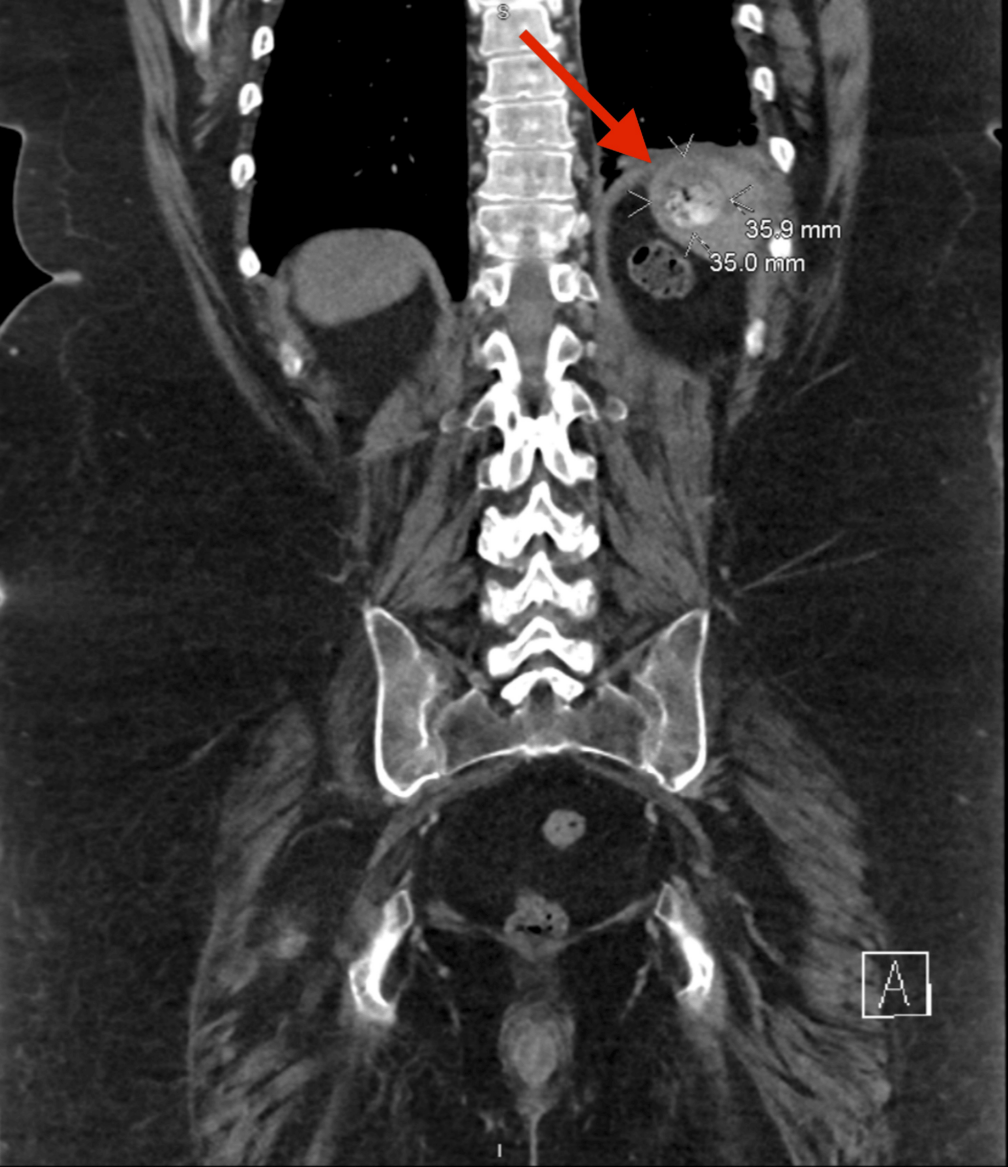
Coronal view of the splenic irritation

A 51-year-old female presented to the emergency department with three hours of acute onset epigastric abdominal pain radiating to the left flank. Her past medical history was significant for Addison’s disease and Sjogren’s disease, requiring long-term treatment with 20 mg of hydrocortisone daily. She also reported associated nausea, chest pain, and shortness of breath. On computed tomography, she had a perforation of the posterior wall of the gastric fundus with involvement of the spleen’s medial aspect. The perforation contained oral contrast, gas locules, and a 1.6-cm radiopaque foreign body with a left-sided reactive pleural effusion (Figures 1-2). Although the patient had a normal white blood cell count of 9.6 on admission, she was tachycardic and tachypneic, and her abdominal exam was significant for rigidity and involuntary guarding. She was subsequently taken to the operating room.

Given the patient’s body habitus, a laparoscopic first approach was performed to facilitate the mobilization of the greater curvature of the stomach up to the level of the hiatus with planned conversion to laparotomy to complete the procedure. After opening, undigested pills were found eroding into the spleen with significant bleeding. Inflammation and friable tissue made local control difficult. Therefore, a splenectomy was performed. A 3-cm perforation of the posterior gastric fundus was also noted. This was repaired primarily in two layers with Vicryl suture (Ethicon, Inc., Somerville, USA) and buttressed with omentum. Following a negative leak test, the abdomen was irrigated and closed in a normal fashion. Her postoperative course was complicated by a leak at the repair site seen with an upper gastrointestinal series with a subsequent return to the operating room for another Graham patch procedure on postoperative day 7, likely due to her need for stress dose steroids in the setting of chronic steroid use. An interventional radiology drainage procedure was also needed for a postoperative abscess. The patient was eventually discharged to a skilled nursing facility.

## Discussion

Gastric perforations are commonly caused by peptic ulcer disease [1]. While most peptic ulcer diseases are caused by NSAID use, less common causes of gastric ulcers include eroded ingested medications [1]. Rarely, foreign bodies that enter the stomach can cause perforation. Gastric contents can spill out of the perforation, causing irritation or frank peritonitis. In the case of foreign body erosion, the object itself can spill out and become a nidus for further problems.

The advent of enteric-coated pills has allowed pills to survive longer within the gastric acid secretions [2]. As the pill survives the acidic environment of the stomach, it can exit through a perforation and irritate nearby structures [3]. As potassium pills are one of the most common radiopaque medications that are ingested, they can show up on a scan [4] within the abdomen. Gastric ulcers have been shown to erode into splenic arteries [5], resulting in pseudoaneurysms [6]. Perforation through the gastric fundus can affect the spleen itself. Erosion of a gastric perforation into the splenic body is rare. This may necessitate splenectomy due to inflammation and friable tissue.

Chronic steroid use has also been shown to increase the rate of gastric perforation by up to 25% [7], which is further increased with concomitant NSAID use with a 2.46 times greater risk [8]. Thinning of the mucosal lining due to increased acid production increases the chances of enteric contents perforating through the stomach lining. In our patient, chronic steroid use due to Addison’s disease necessitated stress dose steroids in the perioperative period. This, in turn, increased the risk of repeat gastric perforation.

Gastrorrhaphy with concomitant splenectomy is rare. Mortality rates of gastric perforation alone range from 5% to 25%, with as high as 50% as age increases [9]. Due to the rarity of concomitant splenectomies, there are no published mortality rates associated with it. Care must be taken to explore the surrounding structures during gastric perforation repair to ensure that enteric contents and foreign bodies are not affecting nearby anatomy. Structures irritated from gastric contents can generally be observed, but consideration should be made for resecting highly vascular structures to prevent postoperative bleeding. Intraoperative findings should ultimately dictate the care of the patient.

## Conclusions

Splenic irritation from intragastric contents is a rare complication of gastric perforation. To assess the extent of enteric content spillage, the entire abdominal cavity must be explored. With the advent of enteric-coated pills, there is an increased likelihood of such pills escaping the stomach and irritating structures around it. Good surgical fundamentals, including thorough exploration of the abdomen and assessment of vascular structures, should be used when operating on such patients.
